# The Impact of High Dose Glucocorticoids on Bone Health and Fracture Risk in Systemic Vasculitides

**DOI:** 10.3389/fendo.2022.806361

**Published:** 2022-02-16

**Authors:** Christopher David Box, Owen Cronin, Barbara Hauser

**Affiliations:** ^1^ Rheumatic Disease Unit, Western General Hospital, Edinburgh, United Kingdom; ^2^ Department of Rheumatology, Bon Secours Hospital Cork, Cork, Ireland; ^3^ School of Medicine, College of Medicine and Health, University College Cork, Cork, Ireland; ^4^ Rheumatology and Bone Disease Unit, Centre for Genomic and Experimental Medicine, Institute of Genetics and Molecular Medicine, University of Edinburgh, Edinburgh, United Kingdom

**Keywords:** vasculitis, osteoporosis, glucococorticoids, bone, fracture risk, fractures, large vessel vasculitis, AAV

## Abstract

Systemic vasculitides are a range of conditions characterized by inflammation of blood vessels which may manifest as single organ or life-threatening multisystem disease. The treatment of systemic vasculitis varies depending on the specific disease but historically has involved initial treatment with high dose glucocorticoids alone or in conjunction with other immunosuppressive agents. Prolonged glucocorticoid treatment is frequently required as maintenance treatment. Patients with small and large vessel vasculitis are at increased risk of fracture. Osteoporosis may occur due to intrinsic factors such as chronic inflammation, impaired renal function and to a large extent due to pharmacological therapy with high dose glucocorticoid or combination treatments. This review will outline the known mechanism of bone loss in vasculitis and will summarize factors attributing to fracture risk in different types of vasculitis. Osteoporosis treatment with specific consideration for patients with vasculitis will be discussed. The use of glucocorticoid sparing immunosuppressive agents in the treatment of systemic vasculitis is a significant area of ongoing research. Adjunctive treatments are used to reduce cumulative doses of glucocorticoids and therefore may significantly decrease the associated fracture risk in patients with vasculitis. Lastly, we will highlight the many unknowns in the relation between systemic vasculitis, its treatment and bone health and will outline key research priorities for this field.

## Introduction

Systemic vasculitides frequently present as acute inflammation of various sized blood vessels which can lead to stenosis and aneurysm of the aorta and its branches in large vessel vasculitis (LVV) or necrosis of arterioles, capillaries and venules in small vessel vasculitis (SVV). Untreated large and small vessel vasculitis can lead to rapid organ damage and consequent threat to life. Hence many conditions require strong immunosuppression most commonly with a prolonged course of high dose Glucocorticoids (GC). Long-term sequelae are frequently a result of acute and chronic inflammation, failure to suppress inflammatory activity or secondary to immunosuppression, in particular GC (1, 2). Osteoporosis and increased fracture risk are known comorbidities of prolonged and high cumulative GC doses (3, 4). It is unclear how much the disease process and the inflammation itself contribute to accelerated bone loss or if the increased fracture risk is mainly a result of the negative impact of GC on bone health and muscle strength. This narrative review will explore the mechanism for rapid bone loss and increased fracture risk in vasculitis, summarize current fracture data in various vasculitis subgroups and outline recent developments which can prevent or mitigate this issue.

## Mechanism of Bone Loss and Increased Fracture Risk in Vasculitis

**Figure 1:**
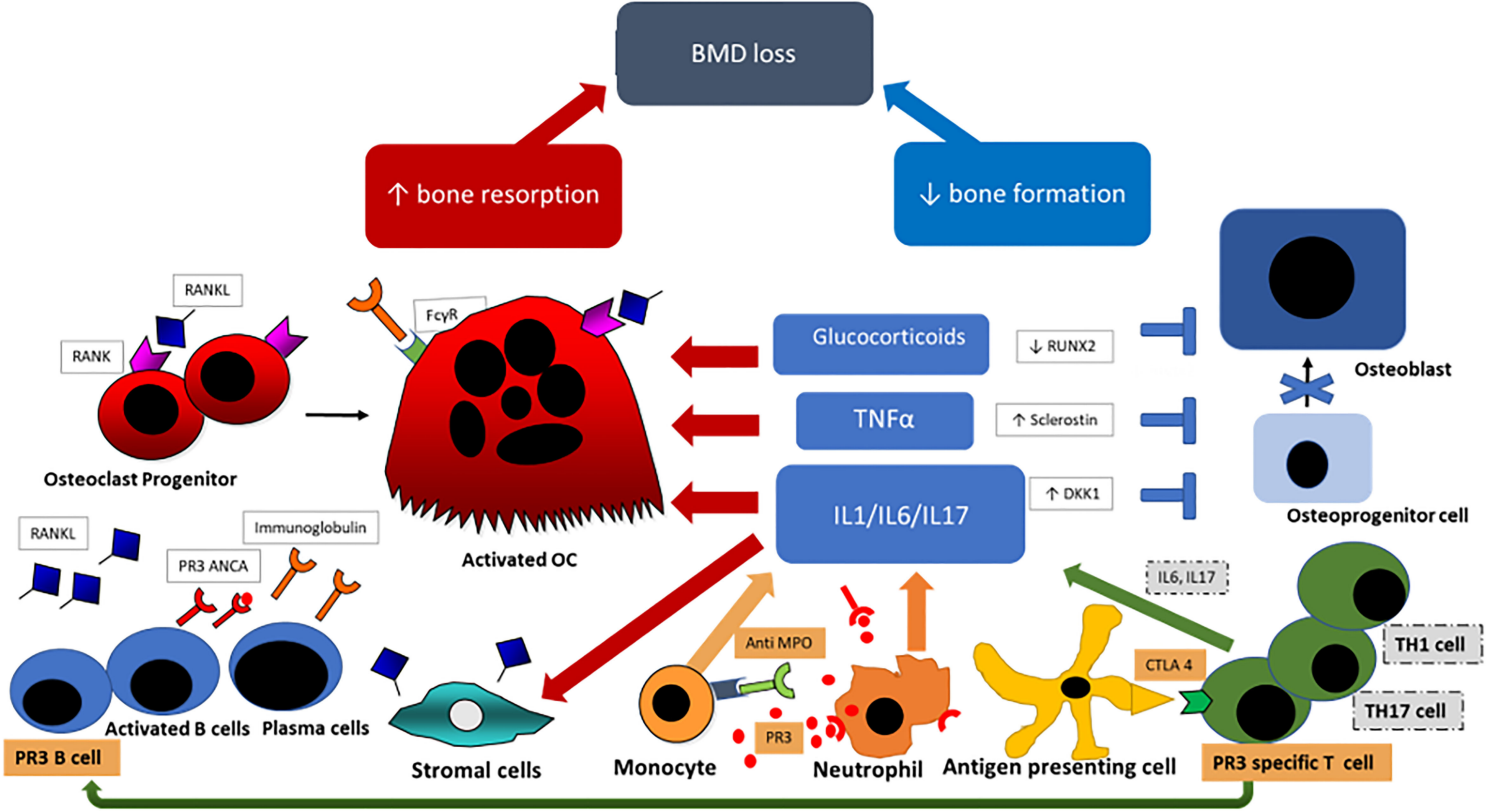
Pathogenesis of bone loss in vasculitis; Anti-neutrophil cytoplasmic antibody (ANCA)-associated vasculitis specific cells and antibodies are highlighted in orange. Primed neutrophils express PR3 [proteinase 3] or MPO [myeloperoxidase] which bind ANCAs and trigger further neutrophil activation and through CD4+ T-lymphocytes stimulation further ANCA production by B-lymphocytes. Key cells and cytokines in the pathogenesis of large vessel vasculitis {LVV} are highlighted in gray. Dendritic cells in the adventitia trigger the inflammatory cascade by activation of T-lymphocytes, predominantly T helper 1 (Th1) and Th17 cells, and express interferon and IL17. Primed neutrophils and Th cells promote proinflammatory cytokine production (Interleukin-6 (IL6), IL1 and Tumour Necrosis Factor (TNF)-alpha) which stimulates osteoclastogenesis through increased RANKL production by stromal cells and through direct osteoclast stimulation. Inflammatory cytokines also inhibit the formation of osteoblasts by increased DKK1 and Sclerostin expression. Glucocorticoids suppress osteoblastogenesis by RUNX2 suppresion and stimulates osteoclast proliferation and longevity. BMD, bond mineral density; RANK4, receptor activator of nuclear factor kappa-B (ligand); PR3, proteinase 3; ANCA, anti-neutrophil cytoplasmic antibody; FcγR, Fc gamma receptor; OC, osteoclast; TNFα, tumuor necrosis factor alpha; IL, interleukin; MPO, myeloperoxidase; RUNX2, runt-related transcription factor 2; DKK1, Dickkopf WNT Signaling Pathway Inhibitor 1; CTLA 4, cytotoxic T-lymphocytes antigen 4; TH1/TH17, T-helper type 1/type 17 cell.

Bone undergoes continuous remodeling and restructuring to maintain its strength and function. In healthy individuals, a precisely coordinated process of bone resorption through osteoclasts and bone formation by osteoblasts allows the repair of damaged bone and replacement of old bone with newly formed mineralized osteoid. Disruption of this remodeling cycle and an increase in bone resorption and/or suppression of bone forming activity leads to systemic bone loss and osteoporosis (5). The most important factors influencing bone turnover in systemic vasculitis are shown in **Figure 1** and discussed in detail below.

### Chronic Inflammation in Vasculitis

In large and small vessel vasculitis the inflammation of vessels is frequently widespread with multisystem involvement and patients usually present with signs of pronounced systemic inflammation (1, 6). The impact of acute or chronic vasculitis on bone physiology is poorly studied. Most data about the interplay between inflammation and bone derives from more common chronic inflammatory conditions such as rheumatoid arthritis (7), spondyloarthritides (8), or connective tissue diseases such as systemic lupus erythematosus (SLE) (9, 10). Inflammatory arthritides and vasculitides have a number of common pathways leading to chronic inflammation with key inflammatory cytokines and cells, supported by the fact that these conditions frequently share some immunosuppressive therapies (11–14). However vasculitides in particular anti-neutrophil cytoplasmic antibody (ANCA)-associated vasculitis (AAV) frequently present with an acute systemic inflammation which can affect multiple organs including kidney, lungs and peripheral nerves, and requires rapid potent immunosuppression including high dose GC in order to prevent severe organ damage and death (15). In contrast, inflammatory arthritides frequently present in an insidious way with polyarthritis as the main manifestation which can be treated initially with mild to moderate immunosuppression and if necessary with subsequent escalation of therapy (16).

#### A) ANCA Associated Vasculitis

Microscopic polyangiitis (MPA) and granulomatosis with polyangiitis (GPA) are ANCA associated vasculitides. AAV are characterized by small-to-medium size blood vessel inflammation and the presence of circulating ANCA antibodies which recognize proteinase 3 (PR3) or myeloperoxidase (MPO). Most GPA patients have ANCA with a cytoplasmic pattern (c-ANCA) that are specific for PR3 whereas in MPA patients ANCA with a perinuclear pattern (p-ANCA) with MPO specificity are frequently found. In AAV, an initial trigger such as infection causes T helper cells to stimulate macrophages, in turn activating neutrophils and leading to formation of neutrophil extracellular traps (NETs) (17–19). The complement system and altered T-lymphocyte homeostasis lead to priming of neutrophils (18, 20, 21). NET degradation is impaired, causing prolonged exposure to NET contents which disrupts tolerance to antigens including PR3 and MPO, leading to ANCA production (19). PR3 and MPO may be expressed on primed neutrophils which bind ANCAs and trigger further excessive neutrophil activation, and both neutrophils and CD4+ T-lymphocytes stimulate further ANCA production by B-lymphocytes, setting up a vicious cycle resulting in proinflammatory cytokine production [Interleukin-6 (IL6), IL8 and Tumour Necrosis Factor (TNF)-alpha (1, 22)] and endothelial damage *via* reactive oxygen species, lytic enzymes and NET components such as histones and matrix metalloproteinases (MMPs) (12, 19, 23–26). The pathogenicity of various immune complexes including PR3 ANCA can be modulated by posttranslational modifications such as glycosylation of immunoglobulins. Genetic associations support a predisposition to AAV or to disease relapse. Examples include patients more commonly expressing specific human leucocyte antigen (HLA) polymorphisms such as HLA-DPB4 or less commonly expressing functional immunoregulatory T-cell receptors such as the cytotoxic T lymphocyte antigen 4 (CTLA) and program death 1 (PD1) (27–29).

#### B) Large Vessel Vasculitis

LVV is characterized by inflammation of the artery wall with predominant CD4+ T-lymphocytes and macrophages which can undergo granulomatous organization in the form of giant cells. In LVV activated dendritic cells in the adventitia can trigger an inflammatory cascade with activation of T-lymphocytes, predominantly T helper 1 (Th1) and Th17 cells, and express interferon and IL17 (30). Dendritic cells drive the inflammatory process and IL1, IL6 and IL21 are highly expressed in giant cell arteritis (GCA) (31, 32).

### Chronic Inflammation and Bone Turnover

Proinflammatory cytokines and their interaction with T- and B-cells propagate chronic inflammation which in turn promotes the differentiation of myeloid cells into macrophages and osteoclasts. The differentiation from multinucleated precursor cells into mature bone resorbing osteoclasts requires the interaction of two crucial cytokines: Macrophage colony-stimulating factor (M-CSF) and Receptor activator of nuclear factor kappa-B ligand (RANKL) (33). Osteoprotegerin (OPG) is a decoy receptor to RANKL and an important regulator of osteoclastogenesis. Mechanisms such as binding of anti-MPO to monocytes or phagocytosis of PR3 expressing neutrophils stimulate the release of inflammatory cytokines including IL1β, IL6, IL8 and TNFα (22, 34). Pro-inflammatory cytokines, particularly IL6 and TNFα, have also been shown to suppress bone formation. Overexpression of TNFα can inhibit osteoblast differentiation either directly through inhibition of Runt-related transcription factor 2 (Runx2) or *via* increased Dickkopf 1 expression which is an important regulator of the Wnt pathway (35–37).

#### A) Large Vessel Vasculitis- Inflammatory Cytokines

The crucial importance of IL6 in the pathogenesis of LVV was confirmed by the success of the introduction of IL6–inhibitors as corticosteroid sparing agents (7, 38). Inflammatory cytokines such as IL1, IL6, IL17 and TNFα can upregulate RANKL production by osteoblasts, T-cells and stromal cells and promote differentiation of osteoclast precursor cells (39) or stimulate osteoclast activity by RANKL independent mechanisms (40, 41). Murine and *in vitro* models have also demonstrated IL6 mediated suppression of osteoblast differentiation which can have a direct impact on skeletal development (42, 43).

#### B) ANCA Associated Vasculitis - the Role of B cells

The clinical success of B-cell depletion in AAV in suppressing disease activity and assuring long term remission provides strong evidence for the important role of B-cells in AAV pathophysiology (44–46).

B cell and bone cell development are closely interlinked.

Stromal cell derived cytokines including RANKL, Osteoprotegerin (OPG) and IL7 are important regulators of osteoclast maturation and differentiation and are also important factors for the development of B cells (47). In murine studies RANK knock out not only resulted in an increased bone mass phenotype (osteopetrosis) but also in impaired lymphocyte development (48).

B cells also produce cytokines which regulate bone cells, in particular RANKL which promotes osteoclastogenesis. Ovariectomy in mice not only causes bone loss through estrogen deficiency and osteoclastic bone resorption but also due to proliferation of RANKL expressing B cells leading to further acceleration of bone resorption (49). In ovariectomized mice lacking B-cells bone loss is attenuated (50).

In particular, activated B cells in the context of chronic inflammation promote bone loss through increased RANKL production and other inflammatory cytokines that promote bone resorption. In addition B cells and in particular plasma cells may influence bone homeostasis through the production of immunoglobulins. In Rheumatoid Arthritis for example immunoglobulins have been shown to directly interact with bone cells, specifically with osteoclasts (51, 52), either *via* the Fcγ receptor on the osteoclast surface (51, 53) or indirectly through blocking OPG (52, 54).

B cell depletion therapy therefore may have a beneficial impact on bone and may prevent accelerated bone loss in chronic inflammatory conditions. To date only a small study of 45 patients with RA who received B cell inhibitor treatment (Rituximab) was performed. After one year of treatment no substantial improvement in BMD was found compared to baseline bone density (55). However this study was likely underpowered and the time frame was too short to detect a significant BMD change. Further studies and particular clinical trials are required to establish the impact of B cell depletion on bone.

### Glucocorticoid Induced Osteoporosis (GIOP) Pathophysiology

GC remain a cornerstone of treatment for most vasculitides and the mainstay of treatment in LVV (56, 57).

The impact of corticosteroids on bone turnover is complex; the most profound effect seems to be on bone formation. Weinstein et al. (58) have shown that chronic GC treatment in mice decreases proliferation of osteoblast precursors and stimulates osteoblast and osteocyte apoptosis, which together leads to a reduction of bone formation. These findings were confirmed on biopsies of patients with GIOP (59). Long-term GC exposure increases expression of the transcription factor peroxisome proliferator-activated receptor (PPAR)γ2 which promotes the differentiation of mesenchymal cells to adipocytes as opposed to osteoblasts. At the same time Runx2, a pivotal transcription factor for osteoblastogenesis, is repressed by GC. GC treatment also has a significant impact on bone resorption. Corticosteroids suppress OPG production (60) which leads to an increase in RANKL/OPG ratio and subsequent stimulation of osteoclast proliferation (59, 60). GC also prolong the lifespan of osteoclasts, further contributing to the imbalance of bone formation and resorption in favour of resorption and hence to net bone loss (58, 61). Therefore, long-term corticosteroid use leads to bone loss and fatty transformation of bone marrow (59, 62, 63).

Extra-skeletal actions of GC on organs such as muscles, kidney and the endocrine system contribute to accelerated bone loss and increased fracture risk. GC decrease calcium absorption in the gastrointestinal tract (64) and decrease the production of sex steroids such as Luteinising hormone (LH), Follicle stimulating hormone (FSH) or Testosterone and Growth hormone (GH) that puts a halt on bone turnover (65). Steroid associated muscle loss (sarcopenia) leads to reduced skeletal loading and postural instability, which is an important risk factor for falls (66).

### Other Medications

Parenteral or oral Cyclophosphamide is frequently used in organ- or life-threatening vasculitis (67, 68). The use of Cyclophosphamide is associated with a number of potential serious side effects including premature ovarian failure characterized by a sharp drop of oestrogens causing early menopause and accelerated bone loss (69). Recently Miyano et al, (70) showed that in an AAV group who sustained fractures, Proton Pump Inhibitor (PPI) users had a higher risk of fractures than histamine-3 receptor antagonist users. Of interest, Abtahi et al. (71) demonstrated in a cohort of patients with rheumatoid arthritis a synergistic effect of GC and PPI in increasing fracture risk. These findings may be of particular importance in patients with GCA and LVV who at disease onset are frequently treated with a combination of high dose GC and PPI.

### Organ Involvement

Acute and chronic renal failure can occur as a consequence of an acute flare of small to medium sized vessel vasculitis (3). Patients with Chronic Kidney Disease (CKD) are at increased risk of osteoporotic fractures (72–74). The mortality associated to fractures increases with worsening renal function (6) and the risk of hip fracture in a population with End Stage Renal Disease (ESRD) is approximately two to four times higher than in the general population (72, 73). The reasons for disturbed bone metabolism in CKD are manifold. Beside accelerated bone loss causing osteoporosis, additional metabolic disorders such as secondary hyperparathyroidism, phosphate retention, elevated fibroblast growth factor -23 (FGF 23), sclerostin overproduction and chronic metabolic acidosis can have a detrimental impact on bone quality. Metabolic bone disorders can result in renal osteodystrophy, adynamic bone disease, osteitis fibrosa or osteomalacia. Additionally, secondary factors such as vitamin D deficiency may increase fracture risk even further (75, 76)

Peripheral neuropathy is one of the frequent long-term sequelae of AAV. A pooled analysis of multiple therapeutic trials showed that 14% of microscopic polyangiitis (MPA) and 22% of granulomatosis with polyangiitis (GPA) patients were found to have developed peripheral neuropathy in long-term outcomes analysis (3). Peripheral neuropathy can lead to gait disorders and increased falls risk (77) which strongly increases fracture risk (78), likely by bone mineral density (BMD) independent mechanism (79). Visual and hearing loss can occur both in LVV and SVV (3, 80) which again substantially increases falls (81) and subsequent fracture risk (82, 83).

### Relative Immobilisation

Clinical manifestations of systemic vasculitis such as mononeuritis multiplex, stroke, blindness or severe arthritis can lead to relative immobility (84–86). A prolonged period of decreased physical activity and chronic inflammation leads to bone loss in addition to an accumulation of visceral fat and sarcopenia (87–89). Recently sarcopenia, measured by reduced hand grip strength, and associated with the type of vasculitis, severity and high C-reactive protein (CRP), seemed to predict increased fracture risk (90). This is in line with previous studies which have shown that change of body composition in form of muscle loss and addition of visceral fat associated with glucocorticoid use increase the risk of osteoporosis and the risk of sustaining fragility fractures (91, 92).

**Figure 2:**
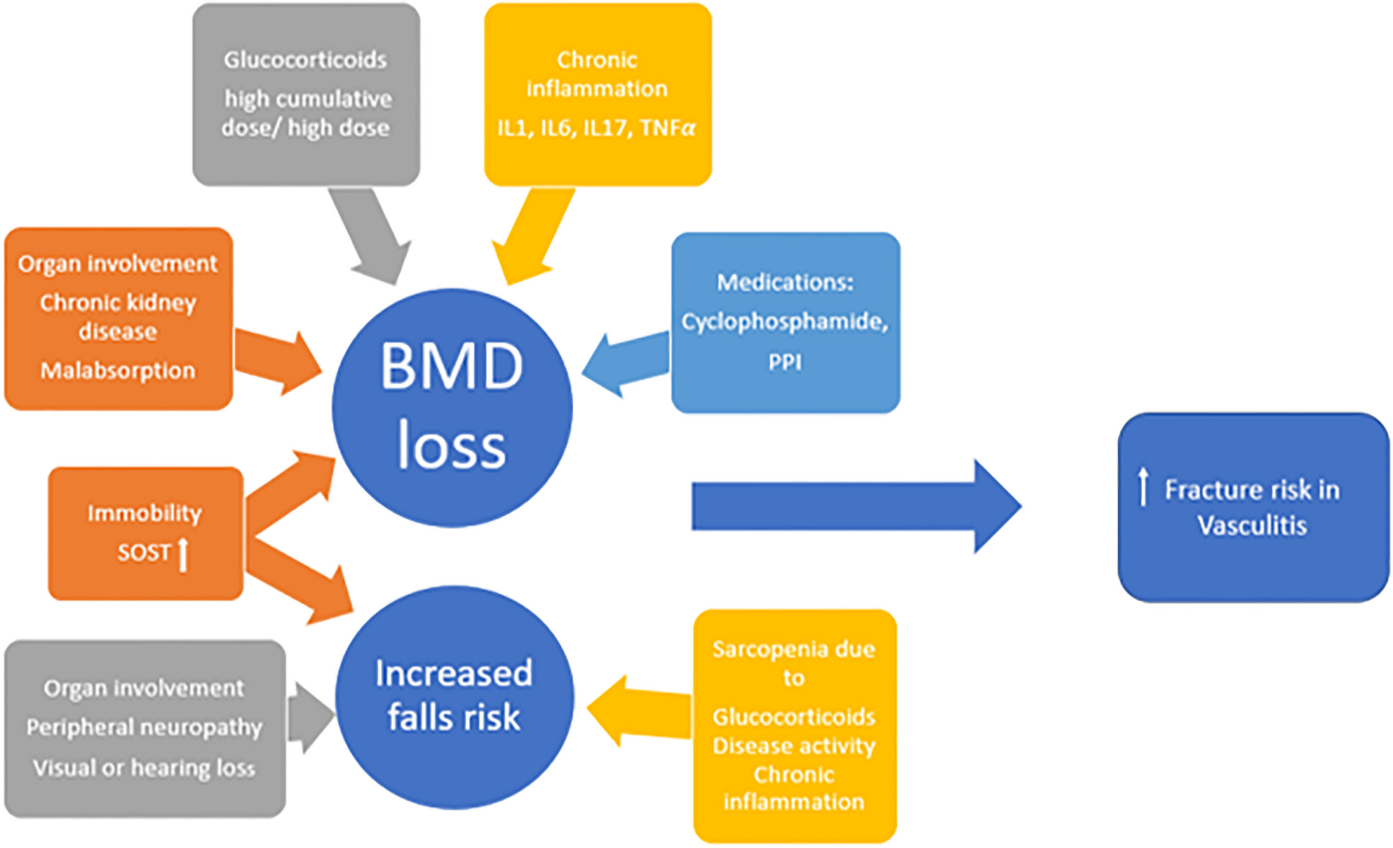
The multifactorial aetiology of increased fracture risk in vasculitides; *IL* interleukin, *TNF* tumour necrosis factor, *PPI* proton pump inhibitor, *SOST* sclerostin, *BMD* bone mineral density.

In summary fracture risk in patients with systemic vasculitides is a composite score of BMD-related and BMD-independent risk factors as shown in **Figure 2**. In order to modify fracture risk many factors, for instance suppression of inflammation, minimizing GC use and avoiding prolonged immobility, should be considered.

## Osteoporosis and Fracture Risk in Different Vasculitis Subgroups

### Giant Cell Arteritis (GCA)/Polymyalgia Rheumatica (PMR)

GCA is the most common primary systemic vasculitis with incidence reported between 1.1 and 43.6 cases per 100,000 in populations aged over 50 years, with significant variation noted geographically (93). PMR is an inflammatory disorder characterized by bilateral upper limb and hip girdle pain and stiffness, with incidence rates of 41 to 112 cases per 100,000 (94–97) among patients over 50 years. GC remain the mainstay of treatment for GCA and PMR. In cohorts of GCA patients, median starting Prednisolone dose was 20-50 mg/day and cumulative doses at 52 weeks were 4000-4800 mg (57). In PMR initial treatment of Prednisolone 15–25 mg is generally followed by a slow taper over 1–2 years (98, 99). Cumulative doses of 3.2 g to 5.4 g are reported (100–102). Treatment beyond 2 years is common, with up to 60% of patients remaining on GC at this point (103).

High rates of osteoporosis are seen in patients with GCA and PMR. Reported prevalence of osteoporosis in GCA varies from 6.25% to as high as 85% (104, 105). The risk of osteoporosis increases over time following diagnosis of GCA and PMR, with the rate of increase highest in the 6 months following diagnosis (105–107). **Table 1** summarises available studies (4, 57, 104, 105, 107–117) on bone health in LVV.

**Table 1:** Summary of studies on osteoporosis and fracture risk in Giant Cell Arteritis (GCA) and Polymyalgia Rheumatica (PMR).

First author	Year	Study population	Age	Details	Level of evidence	Outcome measures	Results
Healey (109)	1996	25 GCA or PMR patients in treatment group 23 GCA or PMR patients in placebo group	71.6	RCT of GC-treated GCA or PMR patients receiving calcium, vitamin D and calcitonin, or receiving calcium, vitamin D and placebo	1b	- Change in BMD at lumbar spine after 2 years - New vertebral fractures at 2 years	- Mean change in lumbar BMD -0.1% intervention group), -0.2% (placebo) - Vertebral fracture incidence 11% and 14% - Higher cumulative GC dose associated with greater loss in BMD
Kermani (107)	2017	204 GCA patients	71.3	Prospective cohort of GCA patients	2b	- Damage items as per Vasculitis Damage Index and LVV Index of Damage	- 22 (10.8%) developed osteoporosis
Petri (104)	2015	4671 GCA patients	N/A	Retrospective cohort of GCA patients (n=4671)	2b	- Incidence of GCA - Cumulative GC dose - Comorbidities associated with GCA	- RR 2.9 for developing osteoporosis after diagnosis of GCA
Mohammad (113)	2017	768 GCA patients 3072 controls	76.1	Retrospective cohort of GCA patients	2b	- Occurrence of osteoporosis or fragility fracture	- RR 2.81 for incident osteoporosis - RR 1.56 for incident fracture
Broder (111)	2016	2497 GCA patients	71	Retrospective cohort of GCA patients	2b	- GC-related adverse events including osteoporosis and fragility fracture	- For every 1g increase in cumulative GC dose, HR 1.05 for osteoporosis and 1.04 for fracture - Osteoporosis rate 0.099 events per person year - Fracture rate 0.066 events per person year
Gale (57)	2018	8777 GCA patients	73	Two retrospective cohorts of GCA patients	2b	- GC cumulative dose - GC-related adverse events - Association of adverse event risk with GC use greater than 52 weeks	- OR of osteoporosis for every 1g increase in cumulative GC dose 1.03-1.06 - OR for fracture for every 1g increase in cumulative GC dose 1.02-1.09 - Risk of osteoporosis for every 1g increase in cumulative GC dose 3-3.4% - Risk of fracture for every 1g increase in cumulative GC dose 1-1.9%
Hatz (105)	1992	47 GCA or PMR patients	N/A	Prospective cohort of GCA and PMR patients	2b	- Side effects attributed to GC at 6 months	- 7 (15.0%) developed osteoporosis within 6 months
Andersson (114)	1990	26 GCA patients	78	Retrospective cohort of GCA patients	2b	- BMD at heel X-ray signs of osteoporosis	- 69% of female patients developed severe spinal osteoporosis after 5 years
Mazzantini (115)	2012	222 PMR patients	71	Retrospective cohort of PMR patients treated with low-dose GC	2b	- Fragility fractures - Osteoporosis	- 55 (24.8%) developed osteoporosis - 31 (14.0%) sustained fragility fractures - GC duration and cumulative dose were significantly associated with osteoporosis and fragility fractures
Sokhal (110)	2021	652 PMR patients	72.4	Prospective cohort of PMR patients	2b	- Fragility fractures at 12 and 24 months	- 72 (11.0%) sustained fragility fracture within 12 months of diagnosis - 60 (9.2%) sustained fragility fracture 12-24 months after diagnosis
Mateo (112)	1993	28 GCA patients 28 PMR patients 48 controls	N/A	Case-control study of patients with GCA, PMR and controls	3b	- BMD at lumbar spine and femoral neck	- Age and cumulative GC dose significant predictors of femoral BMD in men - Age and weight, but not cumulative GC dose, were significant predictors of femoral BMD in women - GCA patients had lower BMD
Wilson (108)	2017	5011 GCA patients 5011 controls	72.9	Retrospective case-control study of GCA patients versus control	3b	- Incidence of osteoporosis or fracture	- IRR for osteoporosis 2.4 in GCA patients - IRR for fracture 1.4 in GCA patients
Paskins (4)	2018	2673 GCA patients 12,136 PMR patients 59,236 controls	71.9	Retrospective case-control study of GCA patients PMR patients	3b	- Time to fracture	- Fracture incidence rate per 10,000 person years 148 for PMR and 147 for GCA - HR for fracture 1.63 for PMR and 1.67 for GCA
Wilson (116)	2017	5011 GCA patients	72.9	Nested case-control studies of GC doses in GCA	3b	- Risk of osteoporosis or fracture associated with increasing GC dose	- 511 (10.2%) developed osteoporosis, mean time to developing osteoporosis 3 years - 408 (8.1%) developed fracture, mean time to fracture 3.2 years - Increased risk of osteoporosis with increasing cumulative GC dose
Haugeberg (117)	2000	GCA or PMR patients - 26 currently treated - 28 previously treated - 30 newly diagnosed	71	Cross-sectional study of BMD in currently treated, previously treated and newly diagnosed GCA or PMR patients	3b	- BMD at radius, spine, hip	- No significant difference in BMD between groups

GC, glucocorticoid; BMD, bone mineral density; RCT, randomized controlled trial; IRR, incidence rate ratio; OR, odds ratio; RR, relative risk; LVV, large vessel vasculitis; HR, hazard ratio.

Higher rates of fractures are seen in both GCA and PMR compared to controls (4, 108) with hazard ratio for fracture 1.63 in PMR and 1.67 in GCA compared to controls. Prospective studies of GCA and PMR patients reveal fragility fracture incidence of 11-14% within 1 to 2 years of diagnosis (109, 110). Rates of fracture correlate with increased cumulative GC doses (4). Evidence from claims data suggests that higher cumulative doses of GC lead to higher complications and increased risk of osteoporosis and fracture, with hazard ratio (HR) for bone-related adverse events increasing 5% for every 1 g increase in cumulative dose of Prednisolone-equivalent GC (111). Similar findings have been established in cohort studies for cumulative doses over 10 g or duration over 2 years (112, 118).

There is some evidence that a lower dose of 5 mg Prednisolone daily can lead to reduced BMD (119), but rates of BMD loss and fracture risk are generally shown to correlate with doses over 10 mg daily of Prednisolone (4, 112).

Few studies have established the risk of osteoporosis attributable to the disease process itself in LVV. Much of the work describing higher rates of osteoporosis in LVV is unable to definitively establish a causative link with GC therapy (120, 121). Rates of osteopenia and osteoporosis are higher in relapsing than newly diagnosed patients with GCA (122), which may relate to higher cumulative doses of GC use but cannot be distinguished from the effect of prolonged inflammation in relapsing cases.

The available data on bone health in LVV typically predate the introduction of the IL6-inhibitor Tocilizumab as a steroid-sparing agent. Adjunctive use of Tocilizumab alongside GC in trials facilitated faster reduction in GC treatment and lower cumulative GC doses in the treatment of GCA (38). Widespread use of Tocilizumab is expected to lead to fewer GC-related adverse events in GCA, including osteoporosis and fractures. However GC alone remains the primary treatment for GCA. BSR and EULAR guidelines recommend Tocilizumab for relapsing patients and those who have already developed, or are at high risk of developing, a complication related to GC (123, 124). The EULAR guideline emphasises that the addition of Tocilizumab must be balanced against the risk of treatment-related adverse effects in comorbid elderly patients. Recent ACR guidance however recommends Tocilizumab plus GC over GC alone for all new patients with GCA (125). As more patients at risk of osteoporosis and fracture are treated with Tocilizumab, the incidence of these outcomes is anticipated to reduce.

### ANCA Associated Vasculitis

AAV is a necrotizing vasculitis that predominantly affects small vessels and is associated with ANCA specific for MPO and/or PR3. AAV mostly present as systemic disease affecting multiple organs. The main clinicopathologic subgroups of AAV are microscopic polyangitis (MPA), granulomatosis with polyangiitis (GPA) and eosinophilic granulomatosis with polyangiitis (EGPA). Although these AAV variants are distinct entities, the clinical manifestations can be overlapping and available data on bone health and fracture risk mostly refers to a pooled AAV group (2, 126). A cross-sectional study (127) showed that amongst 99 AAV patients with an average age of 55 years, 57% had osteopenia and 21% had osteoporosis. Over two thirds (69%) of patients were treated with prolonged high dose GC with an average cumulative dose of 10.7 g. The cumulative GC dose was inversely related to Z-score of lumbar spine and proximal femur confirming the link between high cumulative GC dose and systemic bone loss. In addition to the negative impact of GC on BMD other factors were identified as potential contributors such as low dietary calcium intake and previous cyclophosphamide treatment. This study however was performed almost 20 years ago when the availability, knowledge and use of GC sparing therapies and osteoporosis treatments such as bisphosphonates was scarce.

A population based cohort study from Southern Sweden (128) found that osteoporosis was 4 times more commonly diagnosed in patients with AAV when compared to an age and sex matched general population control cohort (rate ratio 4.6, 95% CI 3.0-7.0). Two long-term follow up studies in SVV including AAV demonstrated that osteoporosis was one of the most commonly reported comorbidities affecting 14-16% of patients when followed up over 7 to 8 years (129). A recent study compared the bone mineral density of 35 treatment naïve AAV patients with 35 healthy, age and sex matched controls. The diagnosis with AAV was associated with osteopenia however when adjusting for other variables such as BMI the association was lost (130).The bone health in newly diagnosed treatment naïve AAV patients is however an interesting question and larger scale studies could provide valuable information on baseline bone status and fracture risk.

Fractures are more common in AAV patients than the general population, with one case control study of 543 AAV patients having twice the risk of hip fracture compared to age and sex matched controls (131). In a retrospective cohort of 22,821 AAV patients, Miyano et al. reported 0.6% developed fractures following diagnosis, with a median time to fracture of 52 days (70). In two further retrospective cohorts of 246 AAV patients 11/246 (4.5%) and 24/278 (8.6%) developed fractures following diagnosis (132, 133), whilst in a cohort of 83 AAV patients aged 65 and over, 8 (9.6%) developed fractures (134).

### Bone Health in Miscellaneous Vasculitic Disorders

Several other forms of small and medium vessel vasculitis can affect children and/or adults [e.g., Behcet’s Disease (BD), Polyarteritis Nodosa (PAN), IgA-associated vasculitis (IgAV)]. These miscellaneous vasculitides are relatively rare, occurring in approximately 1:500,000 people across Europe (135). High doses of GC, often administered to induce remission in the early phases of these rare vasculitides, are highly probable to be detrimental to BMD and fracture risk in affected patients. This is particularly true in these rarer disorders as they often occur in childhood or early adulthood when peak bone mass attainment may not have been achieved.

BD, a multi-system disorder characterized by the presence of recurrent oro-genital inflammation, most commonly occurs between the ages of 20 and 40 years. Typically, it follows a relapsing-remitting course and can affect multiple organ systems. Inflammatory ocular, vascular, neurological or gastro-intestinal disease is associated with a poorer prognosis and usually requires high dose corticosteroid treatment to promptly prevent irreversible end-organ damage. The current literature examining bone health in BD and the impact of corticosteroids is limited. However, two studies have compared BMD between patients with BD and age- and gender-matched healthy controls. Tekin and colleagues investigated differences in BMD and bone turnover markers between 30 patients with BD (mean age 37 years) and 30 healthy controls (mean age 35 years) (136). Lumbar spine and total hip BMD was no different between the two groups and there were no significant differences in markers of bone turnover. Another case-control study by Bicer and colleagues in Turkey compared BMD between patients with BD (n=35) and healthy controls (n=33) (137). This study excluded patients receiving oral corticosteroid therapy and post-menopausal women. Mean age in the BD group was 38 years and in the control group was 40 years. Mean disease duration in the BD group was 6.7 years. Similar to the study by Tekin, BMD was not significantly different between patients with BD and healthy control subjects. The European League Against Rheumatism (EULAR) guidelines for the management of BD advises that if required, high-dose corticosteroids should always be used in combination with concurrent immunosuppressives such as azathioprine, interferonα, or anti-TNFα therapy (138). This ensures that the requirement for long-term high dose corticosteroids in BD is minimized and attenuates the impact of corticosteroids on BMD and fracture risk.

For the management of systemic PAN, the French vasculitis group advise corticosteroid therapy starting at a dose of 1 mg/kg/day of prednisone to a maximum of 60 mg daily (139). There is no agreed or widely accepted reduction strategy and several different regimens are currently being employed worldwide, often for up to 6 or 12 months (140). A prospective study of patients with SVV assessing the long-term outcomes in patients with PAN or MPA identified osteoporosis as one of the three most common sequelae (129). Over a mean follow-up of 98 months, 18% of patients with PAN developed an osteoporotic vertebral fracture compared with 15% of those with MPA highlighting the importance of consideration of bone health in systemic vasculitis.

## Bone Preserving Treatments in Vasculitis: The Role of Steroid-Sparing Therapies

Prevention and management of GIOP is addressed in several guidelines and has been extensively reviewed in other articles and is beyond the scope of this review (141–144). It is worth highlighting that a dual-energy X-ray absorptiometry (DXA) scan for BMD measurement is required in the majority of cases for a fracture risk assessment. As glucocorticoids are particularly associated with osteoporosis of trabecular bone, vertebral fracture assessment (VFA) should be included routinely when DXA scans are performed (145).

Across the spectrum of systemic vasculitis, new, more targeted immunosuppressive and immunomodulatory treatments have been developed to assist with the treatment of systemic vasculitis.

### ANCA Associated Vasculitis Treatment

In AAV, steroid-light and steroid-free regimens are beginning to be used with some success (134, 146). Use of the targeted complement 5a inhibitor Avacopan offers promise as a GC substitute in AAV but more work is required (147). Likewise, Mepolizumab, a monoclonal antibody against IL5 has also demonstrated promise as a treatment adjunct to facilitate greater chances of remission and a faster reduction in GC in EGPA (148, 149).

Publication of the GiACTA study heralded a new era for the treatment of GCA (38). The use of an IL6 inhibitor (Tocilizumab) in GCA has facilitated a significantly more rapid reduction in corticosteroid treatment compared with corticosteroid therapy alone. Significantly the GiACTA trial showed reduced cumulative GC doses by 43.5% and 51.2% in the two arms where Tocilizumab was used alongside GC taper as compared to placebo plus GC taper. Evidence for the glucocorticoid sparing effects of older, more conventional disease modifying immunosuppressants such as methotrexate, azathioprine or mycophenolate mofetil in systemic vasculitis is extremely limited and merits further attention.

## Conclusion

Osteoporosis and fragility fractures are significant long-term complications in vasculitis and most data is available for GCA. High dose GC are undoubtedly one of the main contributing factors. Other factors may increase fracture risk however further research is required to define the role of inflammation, medications and organ involvement on fracture risk in vasculitides. Expansion of non-corticosteroid options for the treatment of systemic vasculitis offers a great hope that in the future, higher fracture rates and impaired bone health will not be a significant problem for our patients suffering from vasculitis.

## Author Contributions

All authors listed have made a substantial, direct, and intellectual contribution to the work, and approved it for publication.

## Funding

We acknowledge the support of NHS Lothian Health Foundation in paying for open access publication fees.

## Conflict of Interest

BH has received a speaker’s fee from Roche.

The remaining authors declare that the research was conducted in the absence of any commercial or financial relationships that could be construed as a potential conflict of interest.

## Publisher’s Note

All claims expressed in this article are solely those of the authors and do not necessarily represent those of their affiliated organizations, or those of the publisher, the editors and the reviewers. Any product that may be evaluated in this article, or claim that may be made by its manufacturer, is not guaranteed or endorsed by the publisher.

## References

[1] Salama AD. Genetics and Pathogenesis of Small-Vessel Vasculitis. Best Pract Res Clin Rheumatol (2018) 32(1):21–30. doi: 10.1016/j.berh.2018.10.002 30526895

[2] Ponte C Águeda AF Luqmani RA. Clinical Features and Structured Clinical Evaluation of Vasculitis. Best Pract Res Clin Rheumatol (2018) 32(1):31–51. doi: 10.1016/j.berh.2018.10.001 30526897

[3] Robson J Doll H Suppiah R Flossmann O Harper L Höglund P. Damage in the Anca-Associated Vasculitides: Long-Term Data From the European Vasculitis Study Group (EUVAS) Therapeutic Trials. Ann Rheumatic Dis (2015) 74(1):177. doi: 10.1136/annrheumdis-2013-203927 24243925

[4] Paskins Z Whittle R Sultan AA Muller S Blagojevic-Bucknall M Helliwell T. Risk of Fracture Among Patients With Polymyalgia Rheumatica and Giant Cell Arteritis: A Population-Based Study. BMC Med (2018) 16(1):4. doi: 10.1186/s12916-017-0987-1 29316928 PMC5761155

[5] Kenkre JS Bassett JHD. The Bone Remodelling Cycle. Ann Clin Biochem (2018) 55(3):308–27. doi: 10.1177/0004563218759371 29368538

[6] Weyand CM Goronzy JJ. Medium- and Large-Vessel Vasculitis. N Engl J Med (2003) 349(2):160–9. doi: 10.1056/NEJMra022694 12853590

[7] Shim J-H Stavre Z Gravallese EM. Bone Loss in Rheumatoid Arthritis: Basic Mechanisms and Clinical Implications. Calcified Tissue Int (2018) 102(5):533–46. doi: 10.1007/s00223-017-0373-1 29204672

[8] Van Mechelen M Gulino GR de Vlam K Lories R. Bone Disease in Axial Spondyloarthritis. Calcified Tissue Int (2018) 102(5):547–58. doi: 10.1007/s00223-017-0356-2 29090349

[9] Güler-Yüksel M Hoes JN Bultink IEM Lems WF. Glucocorticoids, Inflammation and Bone. Calcified Tissue Int (2018) 102(5):592–606. doi: 10.1007/s00223-017-0335-7 29313071

[10] Bultink IEM. Bone Disease in Connective Tissue Disease/Systemic Lupus Erythematosus. Calcified Tissue Int (2018) 102(5):575–91. doi: 10.1007/s00223-017-0322-z PMC5904226 28900675

[11] McInnes IB Schett G. The Pathogenesis of Rheumatoid Arthritis. N Engl J Med (2011) 365(23):2205–19. doi: 10.1056/NEJMra1004965 22150039

[12] Nakazawa D Masuda S Tomaru U Ishizu A. Pathogenesis and Therapeutic Interventions for ANCA-Associated Vasculitis. Nat Rev Rheumatol (2019) 15(2):91–101. doi: 10.1038/s41584-018-0145-y 30542206

[13] Eleftheriou D Brogan PA. Therapeutic Advances in the Treatment of Vasculitis. Pediatr Rheumatol Online J (2016) 14(1):26–. doi: 10.1186/s12969-016-0082-8 PMC4845429 27112923

[14] Lin Y-J Anzaghe M Schülke S. Update on the Pathomechanism, Diagnosis, and Treatment Options for Rheumatoid Arthritis. Cells (2020) 9(4):880. doi: 10.3390/cells9040880 PMC7226834 32260219

[15] Wallace ZS Miloslavsky EM. Management of ANCA Associated Vasculitis. BMJ (2020) 368:m421. doi: 10.1136/bmj.m421 32188597

[16] Aletaha D Smolen JS. Diagnosis and Management of Rheumatoid Arthritis: A Review. JAMA (2018) 320(13):1360–72. doi: 10.1001/jama.2018.13103 30285183

[17] Flint SM McKinney EF Smith KG. Emerging Concepts in the Pathogenesis of Antineutrophil Cytoplasmic Antibody-Associated Vasculitis. Curr Opin Rheumatol (2015) 27(2):197–203. doi: 10.1097/BOR.0000000000000145 25629443

[18] Abdulahad WH Lamprecht P Kallenberg CGM. T-Helper Cells as New Players in ANCA-Associated Vasculitides. Arthritis Res Ther (2011) 13(4):236–. doi: 10.1186/ar3362 PMC3239339 21888687

[19] Nakazawa D Tomaru U Ishizu A. Possible Implication of Disordered Neutrophil Extracellular Traps in the Pathogenesis of MPO-ANCA-Associated Vasculitis. Clin Exp Nephrol (2013) 17(5):631–3. doi: 10.1007/s10157-012-0738-8 23224024

[20] Chavele KM Shukla D Keteepe-Arachi T Seidel JA Fuchs D Pusey CD. Regulation of Myeloperoxidase-Specific T Cell Responses During Disease Remission in Antineutrophil Cytoplasmic Antibody-Associated Vasculitis: The Role of Treg Cells and Tryptophan Degradation. Arthritis Rheum (2010) 62(5):1539–48. doi: 10.1002/art.27403 20155828

[21] Xiao H Schreiber A Heeringa P Falk RJ Jennette JC. Alternative Complement Pathway in the Pathogenesis of Disease Mediated by Anti-Neutrophil Cytoplasmic Autoantibodies. Am J Pathol (2007) 170(1):52–64. doi: 10.2353/ajpath.2007.060573 17200182 PMC1762697

[22] Thieblemont N Witko-Sarsat V Ariel A. Regulation of Macrophage Activation by Proteins Expressed on Apoptotic Neutrophils: Subversion Towards Autoimmunity by Proteinase 3. Eur J Clin Invest (2018) 48(S2):e12990. doi: 10.1111/eci.12990 30039869

[23] Kallenberg CG. Pathogenesis and Treatment of ANCA-Associated Vasculitides. Clin Exp Rheumatol (2015) 33(4 Suppl 92):S11–4. 26457917

[24] Grayson PC Kaplan MJ. At the Bench: Neutrophil Extracellular Traps (NETs) Highlight Novel Aspects of Innate Immune System Involvement in Autoimmune Diseases. J Leukoc Biol (2016) 99(2):253–64. doi: 10.1189/jlb.5BT0615-247R PMC4718195 26432901

[25] Falk RJ Terrell RS Charles LA Jennette JC. Anti-Neutrophil Cytoplasmic Autoantibodies Induce Neutrophils to Degranulate and Produce Oxygen Radicals *In Vitro* . Proc Natl Acad Sci USA (1990) 87(11):4115–9. doi: 10.1073/pnas.87.11.4115 PMC54058 2161532

[26] Nakazawa D Shida H Kusunoki Y Miyoshi A Nishio S Tomaru U. The Responses of Macrophages in Interaction With Neutrophils That Undergo NETosis. J Autoimmun (2016) 67:19–28. doi: 10.1016/j.jaut.2015.08.018 26347075

[27] Osman MS Tervaert JWC. Anti-Neutrophil Cytoplasmic Antibodies (ANCA) as Disease Activity Biomarkers in a “Personalized Medicine Approach” in ANCA-Associated Vasculitis. Curr Rheumatol Rep (2019) 21(12):76. doi: 10.1007/s11926-019-0872-3 31879818

[28] Hilhorst M Arndt F Joseph Kemna M Wieczorek S Donner Y Wilde B. HLA-DPB1 as a Risk Factor for Relapse in Antineutrophil Cytoplasmic Antibody-Associated Vasculitis: A Cohort Study. Arthritis Rheumatol (2016) 68(7):1721–30. doi: 10.1002/art.39620 26866715

[29] Slot MC Sokolowska MG Savelkouls KG Janssen RG Damoiseaux JG Tervaert JW. Immunoregulatory Gene Polymorphisms are Associated With ANCA-Related Vasculitis. Clin Immunol (2008) 128(1):39–45. doi: 10.1016/j.clim.2008.03.506 18448390

[30] Espígol-Frigolé G Corbera-Bellalta M Planas-Rigol E Lozano E Segarra M García-Martínez A. Increased IL-17A Expression in Temporal Artery Lesions is a Predictor of Sustained Response to Glucocorticoid Treatment in Patients With Giant-Cell Arteritis. Ann Rheumatic Dis (2013) 72(9):1481. doi: 10.1136/annrheumdis-2012-201836 22993227

[31] Visvanathan S Rahman MU Hoffman GS Xu S García-Martínez A Segarra M. Tissue and Serum Markers of Inflammation During the Follow-Up of Patients With Giant-Cell Arteritis–a Prospective Longitudinal Study. Rheumatol (Oxford England) (2011) 50(11):2061–70. doi: 10.1093/rheumatology/ker163 PMC3198905 21873264

[32] Hernández-Rodríguez J Segarra M Vilardell C Sánchez M García-Martínez A Esteban MJ. Tissue Production of Pro-Inflammatory Cytokines (IL-1β, Tnfα and IL-6) Correlates With the Intensity of the Systemic Inflammatory Response and With Corticosteroid Requirements in Giant-Cell Arteritis. Rheumatology (2004) 43(3):294–301. doi: 10.1093/rheumatology/keh058 14679293

[33] Jacome-Galarza CE Lee S-K Lorenzo JA Aguila HL. Identification, Characterization, and Isolation of a Common Progenitor for Osteoclasts, Macrophages, and Dendritic Cells From Murine Bone Marrow and Periphery. J Bone Mineral Res (2013) 28(5):1203–13. doi: 10.1002/jbmr.1822 PMC3625454 23165930

[34] O’Brien EC Abdulahad WH Rutgers A Huitema MG O’Reilly VP Coughlan AM. Intermediate Monocytes in ANCA Vasculitis: Increased Surface Expression of ANCA Autoantigens and IL-1β Secretion in Response to Anti-MPO Antibodies. Sci Rep (2015) 5(1):11888. doi: 10.1038/srep11888 26149790 PMC4493694

[35] Diarra D Stolina M Polzer K Zwerina J Ominsky MS Dwyer D. Dickkopf-1 is a Master Regulator of Joint Remodeling. Nat Med (2007) 13(2):156–63. doi: 10.1038/nm1538 17237793

[36] Abbas S Zhang Y-H Clohisy JC Abu-Amer Y. Tumor Necrosis Factor-α Inhibits Pre-Osteoblast Differentiation Through its Type-1 Receptor. Cytokine (2003) 22(1):33–41. doi: 10.1016/S1043-4666(03)00106-6 12946103

[37] Gilbert L He X Farmer P Rubin J Drissi H van Wijnen AJ. Expression of the Osteoblast Differentiation Factor RUNX2 (Cbfa1/AML3/Pebp2αA) Is Inhibited by Tumor Necrosis Factor-α *. J Biol Chem (2002) 277(4):2695–701. doi: 10.1074/jbc.M106339200 11723115

[38] Stone JH Tuckwell K Dimonaco S Klearman M Aringer M Blockmans D. Trial of Tocilizumab in Giant-Cell Arteritis. N Engl J Med (2017) 377(4):317–28. doi: 10.1056/NEJMoa1613849 28745999

[39] Komine M Kukita A Kukita T Ogata Y Hotokebuchi T Kohashi O. Tumor Necrosis Factor-α Cooperates With Receptor Activator of Nuclear Factor κb Ligand in Generation of Osteoclasts in Stromal Cell-Depleted Rat Bone Marrow Cell Culture. Bone (2001) 28(5):474–83. doi: 10.1016/S8756-3282(01)00420-3 11344046

[40] Azuma Y Kaji K Katogi R Takeshita S Kudo A. Tumor Necrosis Factor-α Induces Differentiation of and Bone Resorption by Osteoclasts*. J Biol Chem (2000) 275(7):4858–64. doi: 10.1074/jbc.275.7.4858 10671521

[41] Kudo O Sabokbar A Pocock A Itonaga I Fujikawa Y Athanasou NA. Interleukin-6 and Interleukin-11 Support Human Osteoclast Formation by a RANKL-Independent Mechanism. Bone (2003) 32(1):1–7. doi: 10.1016/S8756-3282(02)00915-8 12584029

[42] De Benedetti F Rucci N Del Fattore A Peruzzi B Paro R Longo M. Impaired Skeletal Development in Interleukin-6–Transgenic Mice: A Model for the Impact of Chronic Inflammation on the Growing Skeletal System. Arthritis Rheumatism (2006) 54(11):3551–63. doi: 10.1002/art.22175 17075861

[43] Kaneshiro S Ebina K Shi K Higuchi C Hirao M Okamoto M. IL-6 Negatively Regulates Osteoblast Differentiation Through the SHP2/MEK2 and SHP2/Akt2 Pathways *In Vitro* . J Bone Mineral Metab (2014) 32(4):378–92. doi: 10.1007/s00774-013-0514-1 24122251

[44] Guillevin L Pagnoux C Karras A Khouatra C Aumaître O Cohen P. Rituximab Versus Azathioprine for Maintenance in ANCA-Associated Vasculitis. N Engl J Med (2014) 371(19):1771–80. doi: 10.1056/NEJMoa1404231 25372085

[45] Pendergraft WF Cortazar FB Wenger J Murphy AP Rhee EP Laliberte KA. Long-Term Maintenance Therapy Using Rituximab-Induced Continuous B-Cell Depletion in Patients With ANCA Vasculitis. Clin J Am Soc Nephrol (2014) 9(4):736. doi: 10.2215/CJN.07340713 24626432 PMC3974359

[46] Alberici F Smith RM Jones RB Roberts DM Willcocks LC Chaudhry A. Long-Term Follow-Up of Patients Who Received Repeat-Dose Rituximab as Maintenance Therapy for ANCA-Associated Vasculitis. Rheumatol (Oxford England) (2015) 54(7):1153–60. doi: 10.1093/rheumatology/keu452 PMC4473766 25477054

[47] Nagasawa T. Microenvironmental Niches in the Bone Marrow Required for B-Cell Development. Nat Rev Immunol (2006) 6(2):107–16. doi: 10.1038/nri1780 16491135

[48] Dougall WC Glaccum M Charrier K Rohrbach K Brasel K De Smedt T. RANK is Essential for Osteoclast and Lymph Node Development. Genes Dev (1999) 13(18):2412–24. doi: 10.1101/gad.13.18.2412 PMC317030 10500098

[49] Kanematsu M Sato T Takai H Watanabe K Ikeda K Yamada Y. Prostaglandin E2 Induces Expression of Receptor Activator of Nuclear Factor–κb Ligand/Osteoprotegrin Ligand on Pre-B Cells: Implications for Accelerated Osteoclastogenesis in Estrogen Deficiency. J Bone Mineral Res (2000) 15(7):1321–9. doi: 10.1359/jbmr.2000.15.7.1321 10893680

[50] Onal M Xiong J Chen X Thostenson JD Almeida M Manolagas SC. Receptor Activator of Nuclear Factor κb Ligand (RANKL) Protein Expression by B Lymphocytes Contributes to Ovariectomy-Induced Bone Loss. J Biol Chem (2012) 287(35):29851–60. doi: 10.1074/jbc.M112.377945 PMC3436192 22782898

[51] Harre U Georgess D Bang H Bozec A Axmann R Ossipova E. Induction of Osteoclastogenesis and Bone Loss by Human Autoantibodies Against Citrullinated Vimentin. J Clin Invest (2012) 122(5):1791–802. doi: 10.1172/JCI60975 PMC3336988 22505457

[52] Hauser B Riches PL Gilchrist T Visconti MR Wilson JF Ralston SH. Autoantibodies to Osteoprotegerin are Associated With Increased Bone Resorption in Rheumatoid Arthritis. Ann Rheumatic Dis (2015) 74(8):1631. doi: 10.1136/annrheumdis-2014-207219 25926154

[53] Koga T Inui M Inoue K Kim S Suematsu A Kobayashi E. Costimulatory Signals Mediated by the ITAM Motif Cooperate With RANKL for Bone Homeostasis. Nature (2004) 428(6984):758–63. doi: 10.1038/nature02444 15085135

[54] Riches PL McRorie E Fraser WD Determann C Rvt H Ralston SH. Osteoporosis Associated With Neutralizing Autoantibodies Against Osteoprotegerin. N Engl J Med (2009) 361(15):1459–65. doi: 10.1056/NEJMoa0810925 19812402

[55] Wheater G Elshahaly M Naraghi K Tuck SP Datta HK van Laar JM. Changes in Bone Density and Bone Turnover in Patients With Rheumatoid Arthritis Treated With Rituximab, Results From an Exploratory, Prospective Study. PloS One (2018) 13(8):e0201527. doi: 10.1371/journal.pone.0201527 30080871 PMC6078302

[56] Guillevin L Mukhtyar C Pagnoux C Yates M. Conventional and Biological Immunosuppressants in Vasculitis. Best Pract Res Clin Rheumatol (2018) 32(1):94–111. doi: 10.1016/j.berh.2018.07.006 30526901

[57] Gale S Wilson JC Chia J Trinh H Tuckwell K Collinson N. Risk Associated With Cumulative Oral Glucocorticoid Use in Patients With Giant Cell Arteritis in Real-World Databases From the USA and UK. Rheumatol Ther (2018) 5(2):327–40. doi: 10.1007/s40744-018-0112-8 PMC6251855 29752705

[58] Weinstein RS Jilka RL Parfitt AM Manolagas SC. Inhibition of Osteoblastogenesis and Promotion of Apoptosis of Osteoblasts and Osteocytes by Glucocorticoids. Potential Mechanisms of Their Deleterious Effects on Bone. J Clin Invest (1998) 102(2):274–82. doi: 10.1172?JCI2799 10.1172/JCI2799 PMC508885 9664068

[59] Hofbauer LC Rauner M. Minireview: Live and Let Die: Molecular Effects of Glucocorticoids on Bone Cells. Mol Endocrinol (2009) 23(10):1525–31. doi: 10.1210/me.2009-0069 PMC5419139 19477950

[60] Kondo T Kitazawa R Yamaguchi A Kitazawa S. Dexamethasone Promotes Osteoclastogenesis by Inhibiting Osteoprotegerin Through Multiple Levels. J Cell Biochem (2008) 103(1):335–45. doi: 10.1002/jcb.21414 17516544

[61] Jia D O'Brien CA Stewart SA Manolagas SC Weinstein RS. Glucocorticoids Act Directly on Osteoclasts to Increase Their Life Span and Reduce Bone Density. Endocrinology (2006) 147(12):5592–9. doi: 10.1210/en.2006-0459 PMC1819400 16935844

[62] Vande Berg BC Malghem J Lecouvet FE Devogelaer JP Maldague B Houssiau FA. Fat Conversion of Femoral Marrow in Glucocorticoid-Treated Patients: A Cross-Sectional and Longitudinal Study With Magnetic Resonance Imaging. Arthritis Rheumatism (1999) 42(7):1405–11. doi: 10.1002/1529-0131(199907)42:7<1405::AID-ANR14>3.0.CO;2-W 10403268

[63] Hartmann K Koenen M Schauer S Wittig-Blaich S Ahmad M Baschant U. Molecular Actions of Glucocorticoids in Cartilage and Bone During Health, Disease, and Steroid Therapy. Physiol Rev (2016) 96(2):409–47. doi: 10.1152/physrev.00011.2015 26842265

[64] Huybers S Naber THJ Bindels RJM Hoenderop JGJ. Prednisolone-Induced Ca2+ Malabsorption is Caused by Diminished Expression of the Epithelial Ca2+ Channel TRPV6. Am J Physiol-Gastrointestinal Liver Physiol (2007) 292(1):G92–G7. doi: 10.1152/ajpgi.00317.2006 16901990

[65] Canalis E Giustina A Bilezikian JP. Mechanisms of Anabolic Therapies for Osteoporosis. N Engl J Med (2007) 357(9):905–16. doi: 10.1056/NEJMra067395 17761594

[66] De Rekeneire N Visser M Peila R Nevitt MC Cauley JA Tylavsky FA. Is a Fall Just a Fall: Correlates of Falling in Healthy Older Persons. The Health, Aging and Body Composition Study. J Am Geriatrics Soc (2003) 51(6):841–6. doi: 10.1046/j.1365-2389.2003.51267.x 12757573

[67] Nachman PH Hogan SL Jennette JC Falk RJ. Treatment Response and Relapse in Antineutrophil Cytoplasmic Autoantibody-Associated Microscopic Polyangiitis and Glomerulonephritis. J Am Soc Nephrol (1996) 7(1):33. doi: 10.1681/ASN.V7133 8808107

[68] Stone JH Merkel PA Spiera R Seo P Langford CA Hoffman GS. Rituximab Versus Cyclophosphamide for ANCA-Associated Vasculitis. New Engl J Med (2010) 363(3):221–32. doi: 10.1056/NEJMoa0909905 PMC3137658 20647199

[69] McDermott AM Miller N Wall D Martyn LM Ball G Sweeney KJ. Identification and Validation of Oncologic miRNA Biomarkers for Luminal A-Like Breast Cancer. PloS One (2014) 9(1):e87032. doi: 10.1371/journal.pone.0087032 24498016 PMC3909065

[70] Miyano S Michihata N Sada K-E Uda K Matsui H Fushimi K. Comparison of Fracture Risk Between Proton Pump Inhibitors and Histamine-2 Receptor Antagonists in ANCA-Associated Vasculitis Patients: A Nested Case–Control Study. Rheumatology (2021) 60(4):1717–23. doi: 10.1093/rheumatology/keaa594 33067623

[71] Abtahi S Driessen JHM Burden AM Souverein PC van den Bergh JP van Staa TP. Low-Dose Oral Glucocorticoid Therapy and Risk of Osteoporotic Fractures in Patients With Rheumatoid Arthritis: A Cohort Study Using the Clinical Practice Research Datalink. Rheumatology (2021). doi: 10.1093/rheumatology/keab548 PMC8996777 34255815

[72] Nitsch D Mylne A Roderick PJ Smeeth L Hubbard R Fletcher A. Chronic Kidney Disease and Hip Fracture-Related Mortality in Older People in the UK. Nephrol Dialysis Transplant (2009) 24(5):1539–44. doi: 10.1093/ndt/gfn678 19075194

[73] Jamal SA Bauer DC Ensrud KE Cauley JA Hochberg M Ishani A. Alendronate Treatment in Women With Normal to Severely Impaired Renal Function: An Analysis of the Fracture Intervention Trial. J Bone Mineral Res (2007) 22(4):503–8. doi: 10.1359/jbmr.070112 17243862

[74] Kim SM Long J Montez-Rath M Leonard M Chertow GM. Hip Fracture in Patients With Non-Dialysis-Requiring Chronic Kidney Disease. J Bone Mineral Res (2016) 31(10):1803–9. doi: 10.1002/jbmr.2862 27145189

[75] Salam SN Eastell R Khwaja A. Fragility Fractures and Osteoporosis in CKD: Pathophysiology and Diagnostic Methods. Am J Kidney Dis (2014) 63(6):1049–59. doi: 10.1053/j.ajkd.2013.12.016 24631043

[76] Miller PD. Chronic Kidney Disease and Osteoporosis: Evaluation and Management. Bonekey Rep (2014) 3:542–. doi: 10.1038/bonekey.2014.37 PMC4078413 24991405

[77] Mayne D Stout NR Aspray TJ. Diabetes, Falls and Fractures. Age Ageing (2010) 39(5):522–5. doi: 10.1093/ageing/afq081 20631405

[78] Lee RH Sloane R Pieper C Lyles KW Adler RA Van Houtven C. Clinical Fractures Among Older Men With Diabetes Are Mediated by Diabetic Complications. J Clin Endocrinol Metab (2018) 103(1):281–7. doi: 10.1210/jc.2017-01593 PMC5761492 29099931

[79] Mohseni M Hosseinzadeh P Civitelli R Eisen S. Effect of Peripheral Neuropathy on Bone Mineral Density in Adults With Diabetes: A Systematic Review of the Literature and Meta-Analysis. Bone (2021) 147:115932. doi: 10.1016/j.bone.2021.115932 33757900 PMC8224476

[80] Chu X Wang D Zhang Y Yin Y Cao Y Han X. Comparisons of Clinical Manifestations and Prognosis Between Giant Cell Arteritis Patients With or Without Sensorineural Hearing Loss: A Retrospective Study of Chinese Patients. Medicine (2019) 98(17):e15286–91. doi: 10.1097/MD.0000000000015286 PMC6831369 31027087

[81] Dhital A Pey T Stanford MR. Visual Loss and Falls: A Review. Eye (2010) 24(9):1437–46. doi: 10.1038/eye.2010.60 20448666

[82] Ivers RQ Norton R Cumming RG Butler M Campbell AJ. Visual Impairment and Hip Fracture. Am J Epidemiol (2000) 152(7):633–9. doi: 10.1093/aje/152.7.633 11032158

[83] Kim SY Lee JK Sim S Choi HG. Hearing Impairment Increases the Risk of Distal Radius, Hip, and Spine Fractures: A Longitudinal Follow-Up Study Using a National Sample Cohort. PloS One (2018) 13(2):e0192820. doi: 10.1371/journal.pone.0192820 29438391 PMC5811044

[84] Boki KA Dafni U Karpouzas GA Papasteriades C Drosos AA Moutsopoulos HM. Necrotizing Vasculitis in Greece: Clinical, Immunological and Immunogenetic Aspects. A Study of 66 Patients. Br J Rheumatol (1997) 36(10):1059–66. doi: 10.1093/rheumatology/36.10.1059 9374922

[85] Salvarani C Brown RD Jr Christianson T Miller DV Giannini C Huston J 3rd. An Update of the Mayo Clinic Cohort of Patients With Adult Primary Central Nervous System Vasculitis: Description of 163 Patients. Med (Baltimore) (2015) 94(21):e738. doi: 10.1097/MD.0000000000000738 PMC4616419 26020379

[86] de Boysson H Boulouis G Aouba A Bienvenu B Guillevin L Zuber M. Adult Primary Angiitis of the Central Nervous System: Isolated Small-Vessel Vasculitis Represents Distinct Disease Pattern. Rheumatol (Oxford) (2017) 56(3):439–44. doi: 10.1093/rheumatology/kew434 27940585

[87] Lilleby V Haugen M Mørkrid L Frey Frøslie K Holven KB Førre O. Body Composition, Lipid and Lipoprotein Levels in Childhood-Onset Systemic Lupus Erythematosus. Scand J Rheumatol (2007) 36(1):40–7. doi: 10.1080/03009740600907881 17454934

[88] Santos MJ Vinagre F Canas da Silva J Gil V Fonseca JE. Body Composition Phenotypes in Systemic Lupus Erythematosus and Rheumatoid Arthritis: A Comparative Study of Caucasian Female Patients. Clin Exp Rheumatol (2011) 29(3):470–6. 21640047

[89] Zerwekh JE Ruml LA Gottschalk F Pak CY. The Effects of Twelve Weeks of Bed Rest on Bone Histology, Biochemical Markers of Bone Turnover, and Calcium Homeostasis in Eleven Normal Subjects. J Bone Miner Res (1998) 13(10):1594–601. doi: 10.1359/jbmr.1998.13.10.1594 9783548

[90] Henriquez S Dunogue B Porcher R Regent A Cohen P Berezne A. Handgrip Strength is a Comorbidity Marker in Systemic Necrotizing Vasculitides and Predicts the Risk of Fracture and Serious Adverse Events. Rheumatol (Oxford) (2020) 59(9):2581–90. doi: 10.1093/rheumatology/kez680 32449923

[91] Wickham CA Walsh K Cooper C Barker DJ Margetts BM Morris J. Dietary Calcium, Physical Activity, and Risk of Hip Fracture: A Prospective Study. BMJ (Clin Res ed) (1989) 299(6704):889–92. doi: 10.1136/bmj.299.6704.889 PMC1837771 2510879

[92] Sayer AA Syddall HE Martin HJ Dennison EM Anderson FH Cooper C. Falls, Sarcopenia, and Growth in Early Life: Findings From the Hertfordshire Cohort Study. Am J Epidemiol (2006) 164(7):665–71. doi: 10.1093/aje/kwj255 PMC2062502 16905644

[93] Sharma A Mohammad AJ Turesson C. Incidence and Prevalence of Giant Cell Arteritis and Polymyalgia Rheumatica: A Systematic Literature Review. Semin Arthritis Rheumatism (2020) 50(5):1040–8. doi: 10.1016/j.semarthrit.2020.07.005 32911281

[94] Gran JT Myklebust G. The Incidence of Polymyalgia Rheumatica and Temporal Arteritis in the County of Aust Agder, South Norway: A Prospective Study 1987-94. J Rheumatol (1997) 24(9):1739–43. 9292797

[95] Doran MF Crowson CS O'Fallon WM Hunder GG Gabriel SE. Trends in the Incidence of Polymyalgia Rheumatica Over a 30 Year Period in Olmsted County, Minnesota, USA. J Rheumatol (2002) 29(8):1694–7. 12180732

[96] González-Gay MA Pina T. Giant Cell Arteritis and Polymyalgia Rheumatica: An Update. Curr Rheumatol Rep (2015) 17(2):6. doi: 10.1007/s11926-014-0480-1 25618572

[97] Crowson CS Matteson EL. Contemporary Prevalence Estimates for Giant Cell Arteritis and Polymyalgia Rheumatica, 2015. Semin Arthritis Rheum (2017) 47(2):253–6. doi: 10.1016/j.semarthrit.2017.04.001 PMC5623160 28551169

[98] Dejaco C Singh YP Perel P Hutchings A Camellino D Mackie S. 2015 Recommendations for the Management of Polymyalgia Rheumatica: A European League Against Rheumatism/American College of Rheumatology Collaborative Initiative. Ann Rheumatic Dis (2015) 74(10):1799–807. doi: 10.1136/annrheumdis-2015-207492 26359488

[99] Dasgupta B Borg FA Hassan N Barraclough K Bourke B Fulcher J. BSR and BHPR Guidelines for the Management of Polymyalgia Rheumatica. Rheumatology (2009) 49(1):186–90. doi: 10.1093/rheumatology/kep303a 19910443

[100] Cimmino MA Salvarani C Macchioni P Gerli R Bartoloni Bocci E Montecucco C. Long-Term Follow-Up of Polymyalgia Rheumatica Patients Treated With Methotrexate and Steroids. Clin Exp Rheumatol (2008) 26(3):395–400. 18578959

[101] Partington RJ Muller S Helliwell T Mallen CD Abdul Sultan A. Incidence, Prevalence and Treatment Burden of Polymyalgia Rheumatica in the UK Over Two Decades: A Population-Based Study. Ann Rheumatic Dis (2018) 77(12):1750–6. doi: 10.1136/annrheumdis-2018-213883 30297332

[102] Gabriel SE Sunku J Salvarani C O'Fallon WM Hunder GG. Adverse Outcomes of Antiinflammatory Therapy Among Patients With Polymyalgia Rheumatica. Arthritis Rheum (1997) 40(10):1873–8. doi: 10.1002/art.1780401022 9336424

[103] Giollo A Rossini M Bettili F Ghellere F Fracassi E Idolazzi L. Permanent Discontinuation of Glucocorticoids in Polymyalgia Rheumatica Is Uncommon But May Be Enhanced by Amino Bisphosphonates. J Rheumatol (2019) 46(3):318–22. doi: 10.3899/jrheum.180324 30385701

[104] Petri H Nevitt A Sarsour K Napalkov P Collinson N. Incidence of Giant Cell Arteritis and Characteristics of Patients: Data-Driven Analysis of Comorbidities. Arthritis Care Res (2015) 67(3):390–5. doi: 10.1002/acr.22429 25132663

[105] Hatz HJ Helmke K. Polymyalgia Rheumatica and Giant Cell Arteritis; Diagnosis and Side Effects of Low-Dose Long-Term Glucocorticoid Therapy. Z Rheumatol (1992) 51(5):213–21. 1476006

[106] Pearce G Ryan PF Delmas PD Tabensky DA Seeman E. The Deleterious Effects of Low-Dose Corticosteroids on Bone Density in Patients With Polymyalgia Rheumatica. Br J Rheumatol (1998) 37(3):292–9. doi: 10.1093/rheumatology/37.3.292 9566670

[107] Kermani TA Sreih AG Cuthbertson D Carette S Hoffman GS Khalidi NA. Evaluation of Damage in Giant Cell Arteritis. Rheumatology (2017) 57(2):322–8. doi: 10.1093/rheumatology/kex397 PMC5850105 29112740

[108] Wilson JC Sarsour K Collinson N Tuckwell K Musselman D Klearman M. Incidence of Outcomes Potentially Associated With Corticosteroid Therapy in Patients With Giant Cell Arteritis. Semin Arthritis Rheum (2017) 46(5):650–6. doi: 10.1016/j.semarthrit.2016.10.001 27839741

[109] Healey JH Paget SA Williams-Russo P Szatrowski TP Schneider R Spiera H. A Randomized Controlled Trial of Salmon Calcitonin to Prevent Bone Loss in Corticosteroid-Treated Temporal Arteritis and Polymyalgia Rheumatica. Calcified Tissue Int (1996) 58(2):73–80. doi: 10.1007/BF02529727 8998681

[110] Sokhal BS Hider SL Paskins Z Mallen CD Muller S. O03 Prevalence of Fragility Fractures and Medication Prescription for Osteoporosis in Patients With Polymyalgia Rheumatica: Results From the PMR Cohort Study. Rheumatology (2021) 60(Supplement_1). doi: 10.1093/rheumatology/keab246.002 PMC8712242 34988356

[111] Broder MS Sarsour K Chang E Collinson N Tuckwell K Napalkov P. Corticosteroid-Related Adverse Events in Patients With Giant Cell Arteritis: A Claims-Based Analysis. Semin Arthritis Rheum (2016) 46(2):246–52. doi: 10.1016/j.semarthrit.2016.05.009 27378247

[112] Mateo L Nolla JM Rozadilla A Rodríguez-Moreno J Niubó R Valverde J. Bone Mineral Density in Patients With Temporal Arteritis and Polymyalgia Rheumatica. J Rheumatol (1993) 20(8):1369–73. 8230021

[113] Mohammad AJ Englund M Turesson C Tomasson G Merkel PA. Rate of Comorbidities in Giant Cell Arteritis: A Population-Based Study. J Rheumatol (2017) 44(1):84–90. doi: 10.3899/jrheum.160249 27803140

[114] Andersson R Rundgren Å Rosengren K Bengtsson BÅ Malmvall BE MellstrÖM D. Osteoporosis After Long-Term Corticosteroid Treatment of Giant Cell Arteritis. J Internal Med (1990) 227(6):391–5. doi: 10.1111/j.1365-2796.1990.tb00177.x 2351926

[115] Mazzantini M Torre C Miccoli M Baggiani A Talarico R Bombardieri S. Adverse Events During Longterm Low-Dose Glucocorticoid Treatment of Polymyalgia Rheumatica: A Retrospective Study. J Rheumatol (2012) 39(3):552–7. doi: 10.3899/jrheum.110851 22247343

[116] Wilson JC Sarsour K Collinson N Tuckwell K Musselman D Klearman M. Serious Adverse Effects Associated With Glucocorticoid Therapy in Patients With Giant Cell Arteritis (GCA): A Nested Case-Control Analysis. Semin Arthritis Rheum (2017) 46(6):819–27. doi: 10.1016/j.semarthrit.p2016.11.006 28040244

[117] Haugeberg G Mykleblast G Dovland H Mikkelsen B Gran JT. No Permanent Reduction in Bone Mineral Density During Treatment of Polymyalgia Rheumatica and Temporal Arteritis Using Low Dose Corticosteroids: A Cross Sectional Study. Scand J Rheumatol (2000) 29(3):163–9. doi: 10.1080/030097400750002030 10898068

[118] Lespessailles E Poupon S Adriambelosoa N Pothuaud L Siroux V Bouillon S. Glucocorticoid-Induced Osteoporosis: Is the Bone Density Decrease the Only Explanation? Joint Bone Spine (2000) 67(2):119–26. doi: 10.1016/S1169-8330(00)80064-2 10769104

[119] Ton FN Gunawardene SC Lee H Neer RM. Effects of Low-Dose Prednisone on Bone Metabolism. J Bone Miner Res (2005) 20(3):464–70. doi: 10.1359/JBMR.041125 15746991

[120] Matteson EL Buttgereit F Dejaco C Dasgupta B. Glucocorticoids for Management of Polymyalgia Rheumatica and Giant Cell Arteritis. Rheum Dis Clin North Am (2016) 42(1):75–90, viii. doi: 10.1016/j.rdc.2015.08.009 26611552

[121] Harris E Tiganescu A Tubeuf S Mackie SL. The Prediction and Monitoring of Toxicity Associated With Long-Term Systemic Glucocorticoid Therapy. Curr Rheumatol Rep (2015) 17(6):513. doi: 10.1007/s11926-015-0513-4 25903665

[122] Tuckwell K Collinson N Dimonaco S Klearman M Blockmans D Brouwer E. Newly Diagnosed vs. Relapsing Giant Cell Arteritis: Baseline Data From the GiACTA Trial. Semin Arthritis Rheum (2017) 46(5):657–64. doi: 10.1016/j.semarthrit.2016.11.002 27998620

[123] Mackie SL Dejaco C Appenzeller S Camellino D Duftner C Gonzalez-Chiappe S. British Society for Rheumatology Guideline on Diagnosis and Treatment of Giant Cell Arteritis. Rheumatology (2020) 59(3):e1–e23. doi: 10.1093/rheumatology/kez672 31970405

[124] Hellmich B Agueda A Monti S Buttgereit F de Boysson H Brouwer E. 2018 Update of the EULAR Recommendations for the Management of Large Vessel Vasculitis. Ann Rheum Dis (2020) 79(1):19–30. doi: 10.1136/annrheumdis-2019-215672 31270110

[125] Maz M Chung SA Abril A Langford CA Gorelik M Guyatt G. 2021 American College of Rheumatology/Vasculitis Foundation Guideline for the Management of Giant Cell Arteritis and Takayasu Arteritis. Arthritis Rheumatol (2021) 73(8):1349–65. doi: 10.1002/art.41774 PMC12344528 34235884

[126] King C Harper L Little M. The Complications of Vasculitis and its Treatment. Best Pract Res Clin Rheumatol (2018) 32(1):125–36. doi: 10.1016/j.berh.2018.07.009 30526892

[127] Boomsma MM Stegeman CA Kramer AB Karsijns M Piers DA Cohen Tervaert JW. Prevalence of Reduced Bone Mineral Density in Patients With Anti-Neutrophil Cytoplasmic Antibody Associated Vasculitis and the Role of Immunosuppressive Therapy: A Cross-Sectional Study. Osteoporosis Int (2002) 13(1):74–82. doi: 10.1007/s198-002-8341-z 11883409

[128] Englund M Merkel PA Tomasson G Segelmark M Mohammad AJ. Comorbidities in Patients With Antineutrophil Cytoplasmic Antibody-Associated Vasculitis Versus the General Population. J Rheumatol (2016) 43(8):1553. doi: 10.3899/jrheum.151151 27252425

[129] Samson M Puéchal X Devilliers H Ribi C Cohen P Bienvenu B. Long-Term Follow-Up of a Randomized Trial on 118 Patients With Polyarteritis Nodosa or Microscopic Polyangiitis Without Poor-Prognosis Factors. Autoimmun Rev (2014) 13(2):197–205. doi: 10.1016/j.autrev.2013.10.001 24161361

[130] Yoo BW Jung SM Song JJ Park YB Lee SW. Prevalence of Osteopenia in Drug-Naive Patients With Antineutrophil Cytoplasmic Antibody-Associated Vasculitis: A Monocentric Study. J Clin Rheumatol (2021) 27(8):e330–e5. doi: 10.1097/RHU.0000000000001413 32530864

[131] Sarica SH Gallacher PJ Dhaun N Sznajd J Harvie J McLaren J. Multimorbidity in Antineutrophil Cytoplasmic Antibody–Associated Vasculitis: Results From a Longitudinal, Multicenter Data Linkage Study. Arthritis Rheumatol (2021) 73(4):651–9. doi: 10.1002/art.41557 33058567

[132] Booth AD Almond MK Burns A Ellis P Gaskin G Neild GH. Outcome of ANCA-Associated Renal Vasculitis: A 5-Year Retrospective Study. Am J Kidney Dis (2003) 41(4):776–84. doi: 10.1016/S0272-6386(03)00025-8 12666064

[133] Gayraud M Guillevin L Le Toumelin P Cohen P Lhote F Casassus P. Long-Term Followup of Polyarteritis Nodosa, Microscopic Polyangiitis, and Churg-Strauss Syndrome: Analysis of Four Prospective Trials Including 278 Patients. Arthritis Rheumatism (2001) 44(3):666–75. doi: 10.1002/1529-0131(200103)44:3<666::AID-ANR116>3.0.CO;2-A 11263782

[134] McGovern D Williams SP Parsons K Farrah TE Gallacher PJ Miller-Hodges E. Long-Term Outcomes in Elderly Patients With ANCA-Associated Vasculitis. Rheumatol (Oxford) (2020) 59(5):1076–83. doi: 10.1093/rheumatology/kez388 PMC7671635 31794032

[135] Scott DG Watts RA. Systemic Vasculitis: Epidemiology, Classification and Environmental Factors. Ann Rheum Dis (2000) 59(3):161–3. doi: 10.1136/ard.59.3.161 PMC1753099 10700420

[136] Tekin NS Ozdolap S Sarikaya S Esturk E Gumustas S. Bone Mineral Density and Bone Turnover Markers of Patients With Behçet's Disease. J Eur Acad Dermatol Venereol (2007) 21(1):25–9. doi: 10.1111/j.1468-3083.2006.01845.x 17207163

[137] Bicer A Tursen U Kaya TI Ozer C Camdeviren H Ikizoglu G. Bone Mineral Density in Patients With Behçet's Disease. Rheumatol Int (2004) 24(6):355–8. doi: 10.1007/s00296-003-0381-5 14556035

[138] Hatemi G Christensen R Bang D Bodaghi B Celik AF Fortune F. 2018 Update of the EULAR Recommendations for the Management of Behçet's Syndrome. Ann Rheum Dis (2018) 77(6):808–18. doi: 10.1136/annrheumdis-2018-213225 29625968

[139] Terrier B Darbon R Durel CA Hachulla E Karras A Maillard H. French Recommendations for the Management of Systemic Necrotizing Vasculitides (Polyarteritis Nodosa and ANCA-Associated Vasculitides). Orphanet J Rare Dis (2020) 15(Suppl 2):351. doi: 10.1186/s13023-020-01621-3 33372616 PMC7771069

[140] Royal College of Physicians and Bone and Tooth Society of Great Britain. Osteoporosis: Clinical Guidelines for Prevention and Treatment: Update on Pharmacological Interventions and an Algorithm for Management2000. London: Royal College of Physicians (2000).

[141] Buckley L Guyatt G Fink HA Cannon M Grossman J Hansen KE. 2017 American College of Rheumatology Guideline for the Prevention and Treatment of Glucocorticoid-Induced Osteoporosis. Arthritis Care Res (Hoboken) (2017) 69(8):1095–110. doi: 10.1002/acr.23279 28585410

[142] Compston J Cooper A Cooper C Gittoes N Gregson C Harvey N. UK Clinical Guideline for the Prevention and Treatment of Osteoporosis. Arch Osteoporos (2017) 12(1):43. doi: 10.1007/s11657-017-0324-5 28425085 PMC5397452

[143] Duru N van der Goes MC Jacobs JWG Andrews T Boers M Buttgereit F. EULAR Evidence-Based and Consensus-Based Recommendations on the Management of Medium to High-Dose Glucocorticoid Therapy in Rheumatic Diseases. Ann Rheumatic Dis (2013) 72(12):1905–13. doi: 10.1136/annrheumdis-2013-203249 23873876

[144] Hayes KN BU Hauser B Burden AM. Winter EM When to Start and Stop Bone-Protecting Medication for Preventing Glucocorticoid-Induced Osteoporosis. Front Endocrinol (2021) 12(1237). doi: 10.3389/fendo.2021.782118 PMC8715727 34975756

[145] Lems WF Paccou J Zhang J Fuggle NR Chandran M Harvey NC. Vertebral Fracture: Epidemiology, Impact and Use of DXA Vertebral Fracture Assessment in Fracture Liaison Services. Osteoporos Int (2021) 32(3):399–411. doi: 10.1007/s00198-020-05804-3 33475820 PMC7929949

[146] Farrah TE Prendecki M Hunter RW Lahiri R Cairns TD Pusey CD. Glucocorticoid-Free Treatment of Severe ANCA-Associated Vasculitis. Nephrol Dial Transplant (2021) 36(4):739–42. doi: 10.1093/ndt/gfaa310 PMC7611118 33367854

[147] Jayne DRW Merkel PA Schall TJ Bekker P. Avacopan for the Treatment of ANCA-Associated Vasculitis. N Engl J Med (2021) 384(7):599–609. doi: 10.1056/NEJMoa2023386 33596356

[148] Wechsler ME Akuthota P Jayne D Khoury P Klion A Langford CA. Mepolizumab or Placebo for Eosinophilic Granulomatosis With Polyangiitis. N Engl J Med (2017) 376(20):1921–32. doi: 10.1056/NEJMoa1702079 PMC5548295 28514601

[149] Bettiol A Urban ML Dagna L Cottin V Franceschini F Del Giacco S. Mepolizumab for Eosinophilic Granulomatosis With Polyangiitis (EGPA): A European Multicenter Observational Study. Arthritis Rheumatol (2021) 20(3):4648–70. doi: 10.1002/art.41943 PMC9305132 34347947

